# Lung and pleura

**DOI:** 10.1186/1470-7330-15-S1-O23

**Published:** 2015-10-02

**Authors:** Stefan Diederich

**Affiliations:** 1 Department of Diagnostic and Interventional Radiology, Marien Hospital, 40479 Düsseldorf, Germany

## Lung

Pulmonary nodules are commonly observed in patients with cancer as well as in patients with no known malignancy particularly in heavy smokers. Most of these nodules are small (less than 8 mm). Even in cancer patients a large proportion of these small nodules are benign [1]. The likelihood of malignancy depends on individual aspects (cancer type, grading, staging, molecular markers etc.), nodule size and risk factors [1]. For example, in a heavy smoker with lung cancer and one additional nodule larger than 8 mm the nodule is more likely to represent a second primary than a solitary metastasis. In a non-smoker with advanced high-grade soft-tissue sarcoma a solitary nodule is more likely to represent a metastasis. In all cancer patients a significant proportion of pulmonary nodules represent benign lesions such as pulmonary lymph nodes or granulomas [2,3].

Non-solid nodules (ground glass opacities) are more likely to represent lung cancer (adenocarcinoma) than solid nodules [4,5]. However, they may represent benign lesions such as focal fibrosis or haemorrhage. Furthermore, some (haemorrhagic) metastases may present as non-solid nodules [6].

Consolidation or diffuse ground glass usually represents benign disease such as pneumonia. However, in adenocarcinoma with predominantly lepidic growth consolidation or diffuse ground glass may be due to cancer spread in the lung.

## Pleura

Pleural effusion may be due to malignant spread (pleural carcinomatosis) or to several benign conditions (heart or renal failure, haemorrhage, infection, etc.). Malignancy is usually confirmed by cytologic analysis of pleura fluid. However, imaging may suggest malignancy if solid pleural lesions are demonstrated within the effusion particularly at the lung base. Unilateral effusion, particularly in the left hemithorax or on the side of the underlying malignancy (e.g. breast, lung cancer) also suggests malignant effusion.

Solid pleural lesions may be clearly benign such as pleural lipoma or calcified pleural plaques in patients with asbestos exposure. Non-calcified focal solid lesions may be benign (e.g. following infection, haemorrhage) or malignant. Although pleural carcinomatosis is usually associated with effusion solid metastases without effusion can occur. Diagnosis usually requires histology [7].
